# Disparity and Inequity of Elementary School Children’s Vision Health and Basic Clinic Resources in Rural and Urban Areas in Taiwan

**DOI:** 10.3390/children13091225

**Published:** 2026-09-10

**Authors:** Jui-Yu Lin, Fu-Gong Lin, Rouh-Mei Hu, Jung-Kai Tseng

**Affiliations:** 1 Department of Optometry, Asia University, Taichung 41354, Taiwan; rueilin@asia.edu.tw (J.-Y.L.); fugong@asia.edu.tw (F.-G.L.); 2 Department of Bioinformatics and Medical Engineering, Asia University, Taichung 41354, Taiwan; rmhu@asia.edu.tw

**Keywords:** childhood myopia, geographic disparities, pediatric vision care, public health policy, vision screening

## Abstract

**Highlights:**

**What are the main findings?**

- Among 10,610 Taiwanese elementary school children, myopia and measurable astigmatism were highly prevalent. Myopia prevalence was generally higher in the upper grades. Children living in rural and especially or extremely remote areas had better uncorrected visual acuity than those living in urban areas. Rural children had lower odds of myopia, whereas children in rural and especially or extremely remote areas had higher odds of measurable astigmatism.
- Optometry clinics were more concentrated in urban areas, and a higher density of optometry clinics was associated with lower odds of myopia.

**What are the implications of the main findings?**

- Better uncorrected visual acuity does not necessarily indicate a lower prevalence or risk of refractive error. Comprehensive vision assessment should therefore include refractive examinations, and school-based screening and referral services should be strengthened, including early screening for high astigmatism.
- Eye-care strategies should be tailored to regional needs. Remote areas may benefit from improved access to optometric services and affordable refractive correction, whereas urban areas may require greater emphasis on lifestyle and environmental interventions related to myopia prevention.

**Abstract:**

**Background/Objectives**: Myopia is a major global public health concern, particularly in East Asia. Taiwan’s exceptionally high prevalence of childhood myopia provides a valuable population model for investigating refractive development and prevention. This study examined geographic disparities in visual acuity, refractive errors, and optometric resources among Taiwanese elementary school children. **Methods**: This retrospective nationwide study analyzed anonymized screening data from 10,610 elementary school children collected from 2014 to 2023. Children were classified as urban, rural, remote, or especially/extremely remote according to governmental criteria. Comprehensive vision examinations were performed by licensed optometrists. Refraction was assessed using autorefraction and retinoscopy without cycloplegia. Refractive errors and healthcare resources were compared across regions using multivariable logistic and linear regression analyses. **Results**: Myopia was present in 86.4% of male and 87.0% of female children, while measurable astigmatism (≥0.25 D) was present in 98.4% of both sexes. High myopia and high astigmatism affected 2.3% and 4.5% of children, respectively. Myopia prevalence varied across grade levels and was 88.9% among sixth-grade children. Compared with urban children, those living in especially or extremely remote areas and rural areas had higher odds of measurable astigmatism (OR = 2.81 and 1.63, respectively; *p* < 0.01). Rural children had significantly lower odds of myopia, while no significant association with myopia was observed among children living in specially or extremely remote areas. Optometry clinics were more concentrated in urban areas, and a higher density of optometry clinics was associated with lower odds of myopia. **Conclusions**: Taiwan provides a valuable model population for understanding geographic, environmental, and healthcare-related factors in childhood myopia. Better visual acuity does not necessarily indicate lower refractive risk. Region-specific strategies should strengthen comprehensive screening and referral, improve optometric access and affordable correction in remote areas, and promote lifestyle and environmental interventions in urban settings.

## 1. Introduction

Myopia is a critical global public health concern; cases are projected to rise from 1.4 billion (22.9%) in 2000 to nearly 5 billion (49.8%) by 2050, with high myopia approaching 1 billion [1]. The problem is particularly acute in East Asia, where the prevalence among school-age children often reaches 80–90%, making myopia an epidemic-level issue [2,3]. Beyond visual impairment, myopia and high myopia increase the risk of severe ocular pathologies such as pathological myopia, glaucoma, cataracts, and macular degeneration [4].

Astigmatism is a common refractive error in children, with prevalence varying by age, ethnicity, region, and diagnostic criteria [5,6]. In Taiwan, it is frequently observed among preschool and school-aged children, predominantly as with-the-rule astigmatism [7]. Clinically significant astigmatism has been associated with amblyopia and deficits in visual acuity, contrast sensitivity, stereoacuity, and other visual functions [8,9]. These findings highlight the importance of school-based vision screening for identifying children with refractive errors who may be at increased risk of visual function abnormalities and require further ophthalmic or optometric evaluation.

In Taiwan, the prevalence of myopia among elementary school children rose significantly between 1983 and 2017 [10]. Urbanization, academic pressure, excessive near work, and screen time increase myopia risk, whereas outdoor activities are protective. Consequently, urban children develop myopia earlier and progress faster than rural peers due to higher academic demands and less outdoor exposure [11,12,13,14]. However, these disparities are not solely environmental; parental myopia and genetic predispositions highlight myopia as a gene–environment interaction disease [15,16,17].

Outdoor activity has consistently shown a protective, dose-dependent effect: an additional hour outdoors per day can reduce myopia risk significantly [18,19,20,21,22]. Yet, its effect on slowing progression among already myopic children remains debated [21]. The COVID-19 pandemic underscored environmental influences, as reduced outdoor time and increased screen exposure accelerated myopia progression in schoolchildren [20,23].

Beyond lifestyle, refractive correction and healthcare access critically influence vision outcomes. Uncorrected refractive error (URE) is a leading cause of low vision and childhood blindness, directly impairing academic performance and mental health [24]. However, disparities in ophthalmic and optometric service distribution hinder timely correction, particularly in rural or socioeconomically disadvantaged areas [25,26,27]. Studies in China and India found large proportions of children with uncorrected myopia and astigmatism, largely due to limited access and low parental awareness [27,28,29]. Similar gaps exist in high-income countries: U.S. children with lapses in health insurance report higher unmet vision needs, underscoring the necessity of sustainable, accessible care [30].

While urban–rural vision disparities are widely studied, few large-scale investigations encompass urban, rural, and remote regions relative to medical resource density. To bridge this gap, we analyzed data from >10,000 Taiwanese elementary students, offering novel evidence on geographic and healthcare-related inequalities to inform future policies and school-based interventions.

## 2. Materials and Methods

### 2.1. Study Design and Population

This retrospective study examined geographic disparities in children’s vision health and primary eye-care resources in Taiwan. A two-stage stratified cluster sampling design was used to ensure adequate geographic coverage and sufficient sample sizes across urban, rural, remote, and outlying areas. Fully anonymized screening data from 10,610 elementary school students were obtained from a vision screening program conducted between 2014 and 2023. The sampling framework was designed primarily to support comparisons across geographic strata rather than proportional population-based sampling or year-by-year surveillance. Approved by a regional university’s Institutional Review Board, the study waived informed consent due to its retrospective and anonymous nature, adhering fully to the Declaration of Helsinki.

The classification of schools in remote areas is officially determined by the Ministry of Education under the Act for Education Development of Schools in Remote Areas and the Standards for the Classification and Recognition of Schools in Remote Areas. Schools are classified into three levels of remoteness: remote, especially remote, and extremely remote. This classification is based on a multidimensional assessment rather than on a single geographic or population-density threshold.

Participants provided basic demographic information, including gender and grade level. Vision health assessments were conducted by qualified, licensed optometrists who performed comprehensive eye examinations using standardized procedures. Refractive errors were evaluated using an autorefractor; however, due to the community-based nature of the screening, cycloplegia was not administered [31]. All measurements were further verified by licensed optometrists using retinoscopy under standardized testing conditions. Myopia was defined as a spherical refraction of −0.50 diopters (D) or lower, high myopia as −5.00 D or lower, hyperopia as +2.00 D or higher, and astigmatism as a cylinder of 0.25 D or higher, with high astigmatism defined as greater than 2.00 D [32,33]. Axial length was measured using an optical biometer, and the presence of strabismus was assessed through cover-uncover and Hirschberg tests.

### 2.2. Statistical Analysis

Descriptive statistics summarized vision parameters and healthcare resources. Refractive error prevalence and resource availability were compared between urban and rural areas using chi-square tests for categorical variables and *t*-tests or ANOVA for continuous variables. Clinic density was assigned by participants’ city of residence. Generalized Estimating Equations (GEE), using an exchangeable correlation structure and robust standard errors, accounted for within-city clustering. Multivariable GEE logistic regression assessed factors associated with refractive errors, adjusting for age, gender, geographic location, and healthcare access, with results reported as ORs and 95% CIs. GEE linear regression analyzed continuous vision outcomes, reporting β coefficients and 95% CIs.

## 3. Results

**Figure 1:**
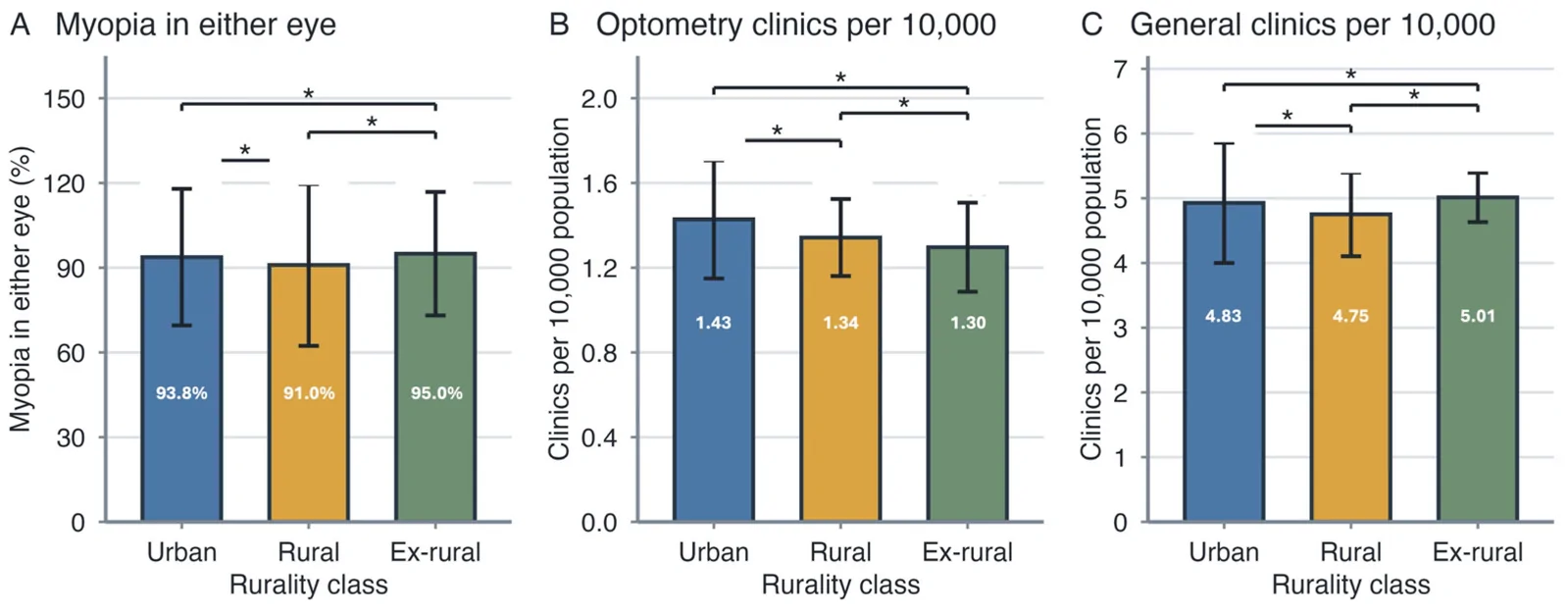
Myopia in either eye and clinic density by rurality class. (**A**) Myopia in either eye prevalence compared with rurality class. (**B**) Optometry clinics density compared with rurality class. (**C**) General clinics density compared with rurality class. * *p* < 0.05.

An analysis was conducted on a cohort of 10,610 elementary school students, with a nearly balanced sex distribution of 47.9% males and 52.1% females. Fifth-grade students accounted for the largest proportion of the sample (24.0%), followed by 3rd grade (18.9%) and 2nd students (17.2%), whereas 1st grade students represented the smallest proportion (12.1%). Regarding residential distribution, 45.3% of participants resided in urban areas, 32.2% in remote areas, and 22.5% in especially or extremely remote areas (Table 1). The prevalence of myopia in at least one eye was 86.4% in males and 87.0% in females, while high myopia was present in 2.5% of males and 2.1% of females. Using the operational threshold of ≥0.25 D, measurable astigmatism was present in 98.4% of both sexes, whereas high astigmatism was present in approximately 4.5%. Myopia prevalence was highest in especially or extremely remote areas (90.4%), followed by urban areas (86.9%) and remote areas (84.3%). Similarly, measurable astigmatism was most prevalent in especially or extremely remote areas (99.2%). In contrast, high astigmatism was most prevalent in urban areas (5.8%), followed by remote areas (3.8%) and especially or extremely remote areas (2.8%) (Table 2, Figure 1A).

The mean uncorrected distance visual acuity (Log MAR VA) exhibited significant variation according to sex, geographic location, and grade level. Female participants demonstrated superior visual acuity compared to their male counterparts (0.042 vs. 0.053; *p* = 0.003). Children residing in urban areas presented with poorer visual acuity, is 0.073, relative to those from rural is 0.040, and especially or extremely remote regions, is 0.008 (*p* < 0.001). Furthermore, visual acuity showed a progressive decline with advancing grade, ranging from 0.007 in 1st grade to 0.102 in 6th grade (*p* < 0.001) (Table 1).

No statistically significant sex differences were observed in the prevalence of myopia, high myopia, measurable astigmatism, or high astigmatism. Significant geographical differences were observed. Myopia prevalence was highest in especially or extremely remote areas (90.4%), followed by urban areas (86.9%) and remote areas (84.3%) (*p* < 0.01). Measurable astigmatism was also most prevalent in especially or extremely remote areas (99.2%) and least prevalent in urban areas (97.9%) (*p* < 0.01). In contrast, high astigmatism was most prevalent in urban areas (5.8%), followed by remote areas (3.8%) and especially or extremely remote areas (2.8%). Myopia prevalence also differed across grade levels, ranging from 80.6% in Grade 1 to 89.6% in Grade 5, with a prevalence of 88.9% in Grade 6. High myopia prevalence varied across grades, ranging from 1.4% in Grade 2 to 3.3% in Grade 6 (Table 2).

Healthcare resource distribution varied across regions. Urban areas had the highest average numbers of optometry and general clinics (259 and 872, respectively), followed by remote (195 and 668) and S- & E-remote areas (170 and 623). Optometry clinic density per 10,000 persons was also highest in urban areas (1.43 ± 0.28), followed by remote (1.34 ± 0.18) and S- & E-remote areas (1.30 ± 0.21). In contrast, general clinic density was highest in S- & E-remote areas (5.01 ± 0.38), followed by urban (4.93 ± 0.93) and remote areas (4.75 ± 0.65).

Logistic regression analyses showed significant geographical differences in refractive errors. Compared with urban children, those living in especially or extremely remote areas had higher odds of measurable astigmatism (OR = 2.81, *p* < 0.01) and lower odds of high astigmatism (OR = 0.47, *p* < 0.01), whereas their odds of myopia did not differ significantly. Children living in remote and rural areas had lower odds of myopia (OR = 0.68, *p* < 0.01), higher odds of measurable astigmatism (OR = 1.63, *p* < 0.01), and lower odds of high astigmatism (OR = 0.64, *p* < 0.01). No significant sex differences were observed for any refractive error. Compared with Grade 1, children in Grades 2–6 had higher odds of myopia, with the highest OR observed in Grade 5 (OR = 1.74, *p* < 0.01). Grade 6 students also had higher odds of high myopia (OR = 1.54, *p* < 0.05). Point estimates for high astigmatism were below 1.0 for Grades 2–6 compared with Grade 1; however, the statistical significance of these associations varied across grades. A higher density of optometry clinics was associated with lower odds of myopia (OR = 0.62, *p* < 0.05), whereas a higher density of general clinics was associated with higher odds of myopia (OR = 2.10, *p* < 0.01) (Table 3; Figure 1B,C).

Multivariable GEE linear regression analysis showed that, compared with children living in urban areas, those living in remote and rural areas (β = −0.032, *p* < 0.001) and especially or extremely remote areas (β = −0.053, *p* < 0.001) had lower distance LogMAR VA values, indicating better visual acuity. Female students also had lower LogMAR VA values than males (β = −0.013, *p* < 0.001). Compared with Grade 1, higher grade levels were associated with higher LogMAR VA values, indicating poorer visual acuity, with the largest difference observed in Grade 6 (β = 0.093, *p* < 0.001). A higher density of optometry clinics was associated with higher LogMAR VA values (β = 0.071, *p* < 0.001), whereas a higher density of general clinics was associated with lower LogMAR VA values (β = −0.024, *p* < 0.001) (Table 4).

## 4. Discussion

Taiwanese elementary school students exhibited a high prevalence of refractive errors, with myopia affecting approximately 86.7% and measurable astigmatism, defined using the study’s operational threshold of ≥0.25 D, present in 98.4%. The high prevalence of myopia is consistent with previous evidence demonstrating a substantial increase in school-age myopia in Taiwan between 1983 and 2017 [10], and with the broader global trend toward increasing childhood myopia [1]. A previous Taiwanese study published in 2004 also reported an association between astigmatism prevalence and myopia [34]. The high prevalence of measurable astigmatism observed in the present study should be interpreted in the context of the relatively sensitive operational threshold of ≥0.25 D. Previous epidemiological studies have used varying thresholds for childhood astigmatism, including ≥0.50, ≥0.75, and ≥1.00 D. Accordingly, the absolute prevalence reported in our study should not be directly compared with estimates derived using more restrictive definitions. Our primary interest was the relative geographic distribution of astigmatic magnitude within a consistently assessed population rather than the estimation of clinically significant astigmatism based on a universal cutoff. Nevertheless, higher degrees of refractive error remain clinically important, as high myopia is associated with ocular complications, while substantial astigmatism in childhood has been associated with amblyopia and impaired visual function [4,8,9].

Therefore, in this study, we examined demographic, geographical, and healthcare-related factors associated with visual outcomes. A notable finding was that schoolchildren in remote areas had better uncorrected visual acuity than their urban counterparts. However, children in remote and rural areas had lower odds of myopia, whereas no significant association with myopia was observed among children living in especially or extremely remote areas. In contrast, children in both remote and rural and especially or extremely remote areas had higher odds of measurable astigmatism. This apparent discrepancy between uncorrected visual acuity and refractive error may reflect factors not directly assessed in the present study, including regional differences in access to refractive correction, spectacle availability, and adherence to spectacle use. From a clinical optometry perspective, refractive errors in underserved areas may be more likely to remain underdiagnosed [29] or uncorrected [28]. Previous studies have suggested that limited healthcare resources and lower parental awareness may contribute to these disparities [25].

This difference between visual performance and refractive error highlights the importance of using multiple indicators to assess children’s visual health.

In addition, high educational pressure [17], long hours of close work, and urban environments with limited outdoor activities are closely related to the development of myopia [13]. It is speculated that these may be the reasons why urban schoolchildren in this study have poorer vision than rural schoolchildren. These data not only highlight the severity of the visual health challenges faced by this group but also highlight the need to develop a comprehensive strategy that combines clinical optometry with public health policies in vision care.

Myopia prevalence differed across grade levels, although the pattern was not monotonic. Because this study was cross-sectional and did not follow the same children over time, these grade-related differences should not be interpreted as temporal progression or as evidence of an emerging myopia epidemic among younger Taiwanese children. Modest differences across grades may also have been influenced by sample composition or sampling variability. Similarly, high astigmatism prevalence varied modestly across grade levels without a clear monotonic trend. This pattern may be consistent with the possibility that high astigmatism is influenced, at least in part, by congenital and early developmental factors [34]; however, this mechanism could not be directly evaluated in the present study. Clinically, early refractive assessment and timely correction may help reduce the risk of visual impairment associated with high astigmatism. From a public health perspective, these findings support the importance of incorporating early vision screening and appropriate refractive assessment into preschool health programs.

While refractive error prevalence did not differ by sex, girls exhibited slightly better visual acuity. Spectacle use and adherence were not recorded in the present study, and the mechanisms underlying this difference therefore could not be determined. Previous work in school-going children has reported that spectacle ownership among those requiring correction is significantly higher in girls than in boys (37.0% vs. 34.7%, *p* < 0.001) [27], and differences in the uptake of optical correction may contribute to the pattern observed here. This possibility remains to be confirmed in studies that directly record spectacle wear and adherence.

Policy-wise, one-size-fits-all interventions cannot address both refractive error prevalence and regional visual inequalities; regionalized, resource-tailored strategies are essential. Remote areas must prioritize enhancing eye care accessibility, strengthen referral systems, and provide affordable corrective lenses. Conversely, since accessibility is not limiting in urban areas, interventions should target behavioral and environmental risk factors—such as increasing outdoor time, optimizing classroom lighting, and limiting screen time to curb myopia progression. Nationally, early screening for high astigmatism should be integrated into preschool health assessments alongside raising awareness of its link to high myopia [19].

An important limitation of this study is the use of non-cycloplegic refraction. Because the data were retrospectively derived from a community-based mass vision screening program, cycloplegic refraction was unavailable in the source dataset. Residual accommodation may therefore have shifted measurements toward more myopic values, potentially overestimating myopia prevalence and underestimating hyperopia. Refraction was initially measured using an autorefractor and subsequently verified by licensed optometrists using retinoscopy. Children with marked discrepancies between autorefraction and retinoscopy were referred for comprehensive ophthalmological examination and excluded from the analytic dataset [31]. Although this approach reduced the likelihood of marked accommodative instability, accommodation-related bias could not be fully eliminated.

The retrospective and cross-sectional design also limits causal inference. Because the sampling framework prioritized adequate coverage across urban, rural, and remote areas rather than proportional population-based sampling, potential selection bias cannot be excluded, and the sample is not nationally representative of all Taiwanese elementary school children. Accordingly, absolute prevalence estimates and their generalizability should be interpreted cautiously. The primary objective, however, was to compare geographic disparities in visual outcomes and eye-care resources rather than estimate national prevalence. Several potential confounders, including lifestyle behaviors, outdoor activity, near-work exposure, parental myopia, and socioeconomic characteristics, were unavailable, and residual confounding remains possible. Since this surveillance screening program did not involve longitudinal follow-up of the same cohort, data were pooled across survey years for the primary spatial analyses. Future population-representative or longitudinal studies using standardized cycloplegic refraction and more comprehensive assessment of potential confounders are warranted.

In summary, this study provides a novel perspective on the relationship between medical resource distribution and vision health outcomes for urban and rural primary school students. It challenges existing assumptions about urban health advantages and highlights the urgent need for specialized care to improve children’s vision health, especially in rural and remote areas. The findings advocate for public health strategies that go beyond simple resource allocation and emphasize the importance of tailored medical interventions to the specific needs of different population groups.

## Figures and Tables

**Table 1:** Vision status distance Log MAR VA.

Variables	*N*	Mean	SD	SE	*p* Value	Tukey After Test
Gender (*N* = 9784)						
Male	4669	0.053	0.188	0.003	0.003	
Female	5115	0.042	0.179	0.003		
Location (*N* = 9708)						
Urban	4401	0.073	0.190	0.003	<0.001	urban > remote
remote	3122	0.040	0.166	0.003		urban > S-, E-remote,
S-, E-remote ^a^	2185	0.008	0.187	0.004		remote > S-, E-remote
Grade (*N* = 9784)						
1st	1214	0.007	0.126	0.004	<0.001	1 < 2, 3, 4, 5, 6
2nd	1732	0.027	0.158	0.004		2 < 4, 5, 6
3rd	1852	0.031	0.175	0.004		3 < 4, 5, 6
4th	1179	0.059	0.188	0.005		4 < 6, 5 < 6
5th	2345	0.057	0.189	0.004		
6th	1462	0.102	0.232	0.006		

*p* values were obtained using one-way ANOVA. ^a^ S-/E-remote includes especially remote and extremely remote areas.

**Table 2:** Refractive error and related variables in terms of spherical power.

	Myopia in Either Eye		High Myopia in Either Eye		Astigmatism in Either Eye (≥0.25 D)		High Astigmatism in Either Eye (>2.00 D)	
	No	Yes	No	Yes	No	Yes	No	Yes
	No. (%)	No. (%)	No. (%)	No. (%)	No. (%)	No. (%)	No. (%)	No. (%)
Gender								
Male	690 (13.6)	4383 (86.4)	4947 (97.5)	126 (2.5)	80 (1.6)	4993 (98.4)	4847 (95.5%)	226 (4.5%)
Female	712 (13.0)	4783 (87.0)	5378 (97.9)	117 (2.1)	89 (1.6)	5408 (98.4)	5250 (95.5%)	247 (4.55%)
Location								
urban	600 (13.1)	3992 (86.9) **	4488 (97.7)	104 (2.3)	98 (2.1)	4495 (97.9) **	4328 (94.2%)	265 (5.8%)
remote	556 (15.7)	2981 (84.3)	3461 (97.9)	76 (2.1)	51 (1.4)	3487 (98.6)	3403 (96.2%)	135 (3.8%)
S- & E-remote ^a^	223 (9.6)	2101 (90.4)	2265 (97.5)	59 (2.5)	18 (0.8)	2306 (99.2)	2259 (97.2%)	65 (2.8%)
Grade								
1st	248 (19.4)	1029 (80.6) **	1246 (97.6)	31 (2.4) *	22 (1.7)	1255 (98.3)	1212 (94.9%)	65 (5.1%)
2nd	299 (16.4)	1521 (83.6)	1794 (98.6)	26 (1.4)	35 (1.9)	1785 (98.1)	1737 (95.4%)	83 (4.6%)
3rd	240 (12.0)	1759 (88.0)	1951 (97.6)	48 (2.4)	28 (1.4)	1972 (98.6)	1915 (95.8%)	85 (4.3%)
4th	172 (13.1)	1140 (86.9)	1282 (97.7)	30 (2.3)	19 (1.4)	1294 (98.6)	1250 (95.2%)	63 (4.8))
5th	264 (10.4)	2277 (89.6)	2487 (97.9)	54 (2.1)	32 (1.3)	2509 (98.7)	2445 (96.2%)	96 (3.8%)
6th	179 (11.1)	1440 (88.9)	1565 (96.7)	54 (3.3)	33 (2.0)	1586 (98.0)	1538 (95.0%)	81 (5.0%)

^a^ S-remote: especially remote; E-remote: extremely remote. * *p* < 0.05, ** *p* < 0.01, tested by Chi-square test.

**Table 3:** Logistic regression analysis primary healthcare and refraction error in children.

	Myopia		High Myopia		Astigmatism		High Astigmatism	
Variables	OR	95% CI	OR	95% CI	OR	95% CI	OR	95% CI
S- & E-remote and rural	0.96	0.76–1.22	1.06	0.78–1.46	2.81	1.66–4.76 **	0.47	0.36–0.64 **
Remote and rural	0.68	0.58–0.81 **	0.92	0.70–1.22	1.63	1.14–2.32 **	0.64	0.50–0.77 **
urban	1		1		1		1	
Female	1.12	0.96–1.30	0.84	0.67–1.06	0.98	0.72–1.33	1	0.83–1.20
Male	1		1		1		1	
Grade 6	1.57	1.19–2.07 **	1.54	1.01–2.36 *	0.86	0.50–1.51	0.9	0.66–1.31
Grade 5	1.74	1.35–2.25 **	1.07	0.70–1.62	1.35	0.77–2.35	0.71	0.52–1.01 **
Grade 4	1.53	1.15–2.04 **	1.12	0.70–1.80	1.17	0.63–2.18	0.93	0.66–1.37
Grade 3	1.48	1.15–1.91 **	1.01	0.65–1.58	1.13	0.64–2.01	0.86	0.61–1.22
Grade 2	1.31	1.02–1.69 *	0.71	0.44–1.16	0.83	0.48–1.43	0.97	0.69–1.37
Grade 1	1		1		1		1	
Optometry clinics Per 10,000	0.62	0.41–0.96 *	0.76	0.36–1.58	0.98	0.37–2.55	0.92	0.77–1.09
General clinics Per 10,000	2.1	1.83–2.41 **	1.12	0.89–1.40	1.16	0.88–1.54	1.24	0.70–2.23

OR = odds ratio; CI = confidence interval. Reference categories were urban area, male, and Grade 1 for geographic location, sex, and grade level, respectively. * *p* < 0.05, ** *p* < 0.01.

**Table 4:** Regression analysis of primary healthcare and children’s vision, dependent variable: distance LogMAR VA.

Variables	Beta	SE	*p* Value
Constant	0.055	0.014	0.000 **
S- & E-remote and rural	−0.053	0.005	0.000 **
Remote and rural	−0.032	0.004	0.000 **
Urban	0		
Female	−0.013	0.004	0.000 **
Male	0		
Grade 6th	0.093	0.007	0.000 **
Grade 5th	0.052	0.005	0.000 **
Grade 4th	0.052	0.007	0.000 **
Grade 3rd	0.031	0.005	0.000 **
Grade 2nd	0.029	0.005	0.000 **
Grade 1st	0		
Optometry clinics Per 10,000	0.071	0.011	0.000 **
General clinics Per 10,000	−0.024	0.004	0.000 **

S-remote: especially remote; E-remote: extremely remote. Reference categories were urban area, male, and Grade 1 for geographic location, sex, and grade level, respectively. ** *p* < 0.01.

## Data Availability

The data that support the findings of this study are available from the International Lions Club Vision Care Network Project Committee, but restrictions apply to the availability of these data, which were used under license for the current study, and so are not publicly available. Data are, however, available from the authors upon reasonable request and with permission of the Vision Care Network.
